# The State of the Art of Natural Polymer Functionalized Fe_3_O_4_ Magnetic Nanoparticle Composites for Drug Delivery Applications: A Review

**DOI:** 10.3390/gels9020121

**Published:** 2023-02-01

**Authors:** Abu Hassan Nordin, Zuliahani Ahmad, Siti Muhamad Nur Husna, Rushdan Ahmad Ilyas, Ahmad Khusairi Azemi, Noraznawati Ismail, Muhammad Luqman Nordin, Norzita Ngadi, Nordin Hawa Siti, Walid Nabgan, Abd Samad Norfarhana, Mohammad Saifulddin Mohd Azami

**Affiliations:** 1 Faculty of Chemical and Energy Engineering, Universiti Teknologi Malaysia, Skudai 81310, Johor, Malaysia; abuhassannordin@gmail.com (A.H.N.); norzita@cheme.utm.my (N.N.); farahfarhana.as@gmail.com (A.S.N.); 2 Faculty of Applied Sciences, Universiti Teknologi MARA (UiTM), Arau 02600, Perlis, Malaysia; zuliahani@uitm.edu.my (Z.A.); husna.md94@gmail.com (S.M.N.H.); saifulddin@uitm.edu.my (M.S.M.A.); 3 Centre for Advanced Composite Materials (CACM), Universiti Teknologi Malaysia (UTM), Skudai 81310, Johor, Malaysia; 4 Institute of Marine Biotechnology, Universiti Malaysia Terengganu, Kuala Terengganu 21030, Terengganu, Malaysia; madkucai89@gmail.com; 5 Department of Clinical Studies, Faculty of Veterinary Medicine, Universiti Malaysia Kelantan, Pengkalan Chepa, Kota Bharu 16100, Kelantan, Malaysia; luqman.n@umk.edu.my; 6 Centre for Nanotechnology in Veterinary Medicine (NanoVet), Faculty of Veterinary Medicine, Universiti Malaysia Kelantan, Pengkalan Chepa, Kota Bharu 16100, Kelantan, Malaysia; 7 Pharmacology Unit, School of Basic Medical Sciences, Faculty of Medicine, Universiti Sultan Zainal Abidin, Kuala Terengganu 20400, Terengganu, Malaysia; hawanordin@gmail.com; 8 Departament d’Enginyeria Química, Universitat Rovira I Virgili, Av. Països Catalans 26, 43007 Tarragona, Spain; wnabgan@gmail.com; 9 Department of Petrochemical Engineering, Politeknik Tun Syed Nasir Syed Ismail, Pagoh Education Hub, Pagoh Muar 84600, Johor, Malaysia

**Keywords:** natural polymer, magnetic nanoparticles, drug delivery, plant, animal, microbe, composites

## Abstract

Natural polymers have received a great deal of interest for their potential use in the encapsulation and transportation of pharmaceuticals and other bioactive compounds for disease treatment. In this perspective, the drug delivery systems (DDS) constructed by representative natural polymers from animals (gelatin and hyaluronic acid), plants (pectin and starch), and microbes (Xanthan gum and Dextran) are provided. In order to enhance the efficiency of polymers in DDS by delivering the medicine to the right location, reducing the medication’s adverse effects on neighboring organs or tissues, and controlling the medication’s release to stop the cycle of over- and under-dosing, the incorporation of Fe_3_O_4_ magnetic nanoparticles with the polymers has engaged the most consideration due to their rare characteristics, such as easy separation, superparamagnetism, and high surface area. This review is designed to report the recent progress of natural polymeric Fe_3_O_4_ magnetic nanoparticles in drug delivery applications, based on different polymers’ origins.

## 1. Introduction

Drug delivery refers to methods, formulations, technologies, and systems for delivering drugs to the body as required to effectively and safely produce their intended therapeutic effects [1]. Active medication molecules should concentrate precisely and specifically in the illness region over an extended time with great controllability to increase their therapeutic effectiveness and decrease unwanted adverse effects. Despite significant advancements in drug delivery formulation over the last two decades, regulating drug entry into the body remains challenging. This is because some drugs need excellent distribution in order to provide the quickest reaction [2].

Therefore, therapeutics have been integrated into polymers to safely transport medications across hostile physiological areas. The incorporated polymers must be biodegradable to enhance release kinetics and avoid carrier accumulation [3]. Biodegradable polymers are often divided into two categories, which are synthetic and natural. Natural polymers have been used to deliver drugs because they have minimal immunogenicity and antibacterial activity while delivering bioactive substances to specific tissues, cells, and cell compartments [4,5,6]. They are readily available and can be extracted mainly from plants [7], animals [8], and microbes [9]. It is worth mentioning that, despite several pre-clinical experiments with a variety of biodegradable polymers showing efficacy, only a small number of these materials have been approved for use in humans and have progressed to post-clinical testing. Then, much work has gone into creating new functional polymers, which offer various benefits over nonfunctionalized polymers in drug delivery systems [10]. Some specific functionalized polymers, called smart polymers, have gained popularity for their ability to react deftly to environmental cues, including temperature [11], pH [12], light [13], electrical fields [14], and magnetic fields [15]. 

Polymeric magnetic composites have emerged as one of these materials with the highest promise because of their particular characteristics, such as rapid response and remote control capabilities [16]. The incorporation of Fe_3_O_4_ magnetic nanoparticles (MNPs) and polymers will result in polymers with magnetic sensitivity, which can address the constraints of conventional polymers in targeted drug administration and remotely controlled release. This paper aims to give readers an overview of the recent development of natural polymeric magnetic composites in drug delivery applications. The first part comprises a special focus on the most commonly used natural polymers derived from animals, plants, and microbes in drug delivery applications. In particular, we emphasized and addressed the latest research and uses of gelatin, hyaluronic acid, pectin, starch, xanthan gum, and dextran polymers in drug delivery systems. Then, this work discusses the potential advantages of using Fe_3_O_4_ MNPs conjugated with natural polymers by the formation of composites and loaded with different drugs for various disease treatments. The magnetic properties of the polymeric Fe_3_O_4_ MNPs composites are useful because they can be directed to the target site via an external magnetic field (EMF). Finally, the future prospects of the research have also been proposed for the advancement of biomedical fields. 

## 2. Applications of Natural Polymers in DDS

**Figure 1:**
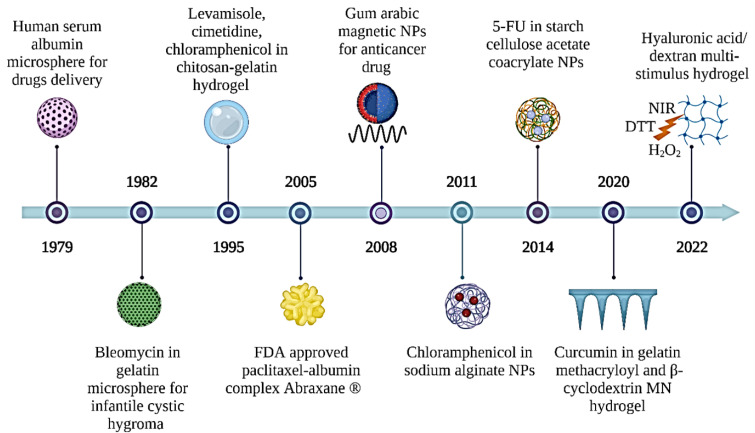
A brief history of the development of natural polymer applications in drug delivery systems. 5-FU: 5-fluorouracil; DTT: dithiothreitol; FDA: food drug administration; NIR: near infrared radiation; NPs: nanoparticles.

**Figure 2:**
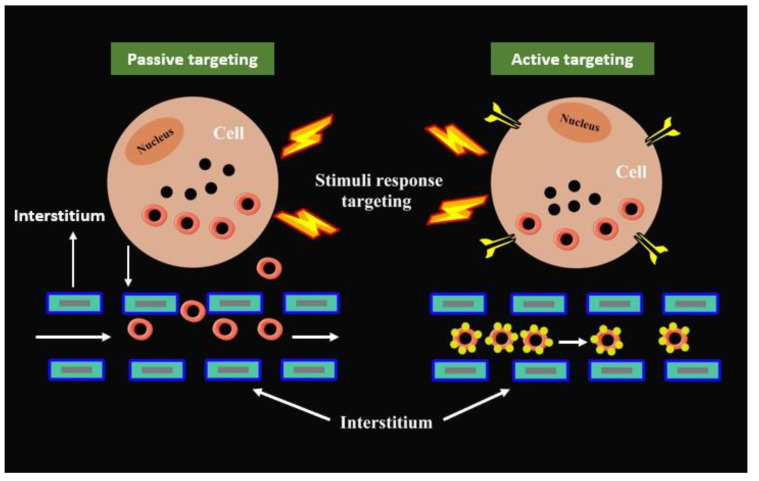
General mechanism of delivering drugs to the targeted cell by natural polymer.

Initially, natural polymers were created for use in a variety of biomaterials applications. In this section, the main focus is to review the development of natural polymers, specifically gelatin, hyaluronic acid, pectin, starch, xanthan gum, and dextran polymers for drug delivery applications. Figure 1 shows a brief history of the development of these natural polymer applications in drug delivery systems (DDS). The illustration of the mechanism of delivering drugs to the targeted cell by natural polymers is presented in Figure 2. Generally, blood flow and the extravasation effect (named passive targeting) are the main mechanisms used by most polymer carriers for cell targeting, which depends on blood circulation [17]. On the other hand, active targeting by polymeric systems is accomplished by coupling ligands that are recognized by certain receptors overexpressed on the cell [18]. Besides that, the polymeric systems may target the cell via the use of stimuli-responsive carriers, intracellular drug targeting, intratumoral drug targeting, and cell vasculature drug targeting [19].

### 2.1. Gelatin

Gelatin is hydrophilic in nature and derived from the controlled denaturation of protein or collagen hydrolysis obtained from animal tissues (e.g., cartilage, skin, and bone) of animals such as porcine, bovine, or fish [20]. The different types and ages of animals, as well as the collagen type such as type I or type II, will affect the physical and chemical characteristics of the extracted collagen [21]. A number of functional side groups in gelatin allow an appropriate mechanical property via chemical crosslinking [22]. Cell–biomaterial interactions of gelatin have shown effectiveness through the exposure of various ligands, such as peptide motifs of Arg-Gly-Asp peptides (RGD) that promote the binding of cells and carriers [21,23]. 

**Figure 3:**
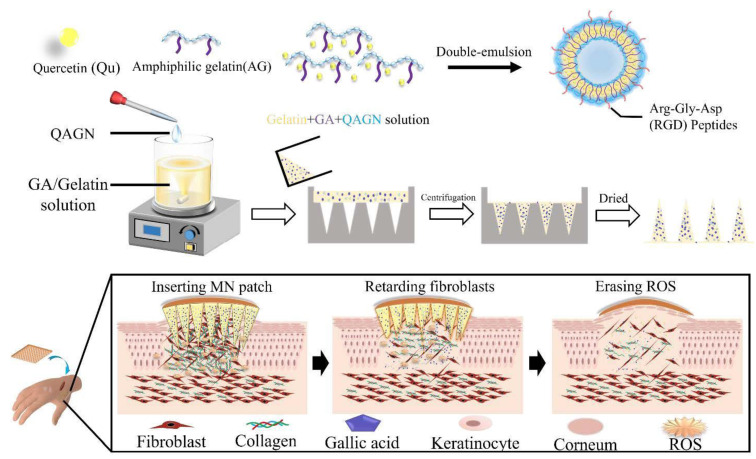
Schematic representation of the amphiphilic gelatin nanocarrier loaded with quercetin preparation and the mechanism of drug release. Keloid scarring can be treated by the transdermal delivery of drug combinations (Qu and gallic acids) via actions of gelatin-based MN composite heterogeneously. Adapted from Chen et al. [22].

Gelatin-based hydrogel involvement in transdermal drug delivery through microneedle (MN) patches allows the delivery of drugs directly into subcutaneous tissue with fewer side effects and is painless. Figure 3 shows a preparation of gelatin-based hydrogel by a recent study on keloid scarring using a gelatin hydrogel-based MN of amphiphilic gelatin nanoparticles loaded with gallic acid and quercetin known as QAGN [22]. The results showed that the MN patches could adhere to the stratum corneum, producing a controlled release of QAGN drugs and downregulated Col I and Col III gene expression of fibroblasts in the combination drug system.

Curcumin, an anticancer drug, is known as an unstable and less soluble drug [24]. A few attempts have been made to enhance its stability and solubility using gelatin polymer in various forms. A hydrogel-based MN fabricated from gelatin methacryloyl and β-cyclodextrin (GelMA-β-CD) loaded with curcumin is introduced to overcome the limitations [25]. MN arrays on 3D B16F10 melanoma spheroids were assessed for in vitro anticancer efficacy, and higher therapeutic efficacy compared to non-transdermal patches. In vivo analysis was conducted to verify the degradability and compatibility of the MN arrays patch of GelMA-β-CD. Another application was presented by formulating curcumin into the gelatin hydrophilic network and hydroxyapatite nanoparticles [26]. This formulation showed a sustained release of curcumin, demonstrating higher internalization into the cell and toxicity towards A549 cells (lung cells) than free curcumin. Both studies on curcumin showed the same results through improvement in the stability and solubility of the curcumin and gelatin polymer, a good biomaterial to be included in the formulation. Another application of natural polymer gelatin is as a drug delivery carrier of carvedilol (CAR) (i.e., a drug used to treat respiratory disorders that possess limitations in terms of solubility and bioavailability) [27]. To overcome these weaknesses, CAR was packed into halloysite nanotubes (HNTs) and was capsulated in a gelatin-based microsphere that was responsive to internal stimuli pHs (HNTs/CAR@GM) [27]. The results showed HNTs/CAR@GM having fast drug release under acidic conditions (pH = 1.2) and non-toxicity against Caco-2 cells. In summary, this study showed that HNTs/CAR@GM showed potential to be exploited in oral DDSs. 

Transdermal drug delivery can be achieved by incorporating drugs of interest into a hydrogel (i.e., crosslinked polymeric networks that are capable to sustain high water amounts). The above studies represent good approaches to gelatin usage in making hydrogel benefiting from its high solubility and amphoteric behavior. MN hydrogels serve as a novel method for transdermal drug delivery with less pain than percutaneous administration, and gelatin serves as a promising natural polymeric material for this system. The summary of studies on gelatin-based DDSs is presented in Table 1.

### 2.2. Hyaluronic Acid (HA)

HA is an anionic polymer of naturally occurring mucopolysaccharide, non-sulfated glycosaminoglycan commonly found in various body parts (e.g., vitreous humor, joints, connective tissue, umbilical cord, and skin) [28,29]. HA comprises N-acetyl-D-glucosamine and D-glucuronic acid that are linked together by glycosidic bonds of β-(1,4) and β-(1,3) [30]. CD44 is a protein surrounded by a membrane that is often highly expressed in various cancer cells [31] and a major receptor for HA. Therefore, HA can target CD44 targeted signaling [32] and is a promising candidate for polymer in delivering anti-cancer drugs to target specific tumor sites. 

Extensive research was conducted on HA in targeting CD44 in various cancer cells. Conjugation of HA-tetraphenyl ethylene (HA-SS-TPE) with glutathione-responsiveness, a novel DDS was designed. This system was developed by self-assembling HA-SS-TPE and loaded with doxorubicin (DOX) to create DOX-loaded polymeric micelles [33]. Interestingly, this novel DDS showed great efficacy in unloading DOX through fast glutathione-triggered dissociation. CD44-positive cells (ES2 and Hela) exhibited a greater intercellular release ratio of DOX compared to CD44-negative cells. The above results showed the great system capability to be incorporated in overexpressed CD44 cancer cells. In addition, an avant-garde halloysite nanotube-based DDS was designed with an HA-modified halloysite (HNTs-NH-HA) compound loaded with DOX [34]. HNTs-NH-HA/DOX increased the DOX therapeutic efficacy and showed high antitumor efficiency in CD44-positive Hela cells compared to HNTs/DOX or free DOX. This concluded that added HA in the system effectively improved DOX targeting, and this serves as a new possibility in cancer treatment. 

DOX-loaded micelle-like nanoparticles for targeted DDS were created by self-assembling conjugate HA–human serum albumin (HAssHSA) attained by covalent attachment of HSA to a cystamine-modified HA [35]. The efficacy of this system is demonstrated by higher cytotoxicity of MDA-MB231 cells conducted by the system compared to free DOX. CD44-mediated internalization of nanoparticles was also confirmed; thus, this system serves as a safety strategy for DOX delivery. HA has been constructed by MN loaded with the drug minoxidil (MXD) for alopecia therapy [36]. In vivo trial was conducted on alopecia mice, and the results showed enhancement of hair dermal papilla (HDP) cells facilitated by a cluster of distinction CD44 and serine-threonine kinase (Akt) phosphorylation. Thus, reduced hair loss in alopecia indicated the effectiveness of delivering MXD using MXD-HA-MNs with minimized side effects of MXD. This study was the first to report the explicit anti-alopecia effects of using MXD-HA-MNs. Next, the HA hydrogel interpenetrating network (IPN) of HA/Poloxamer 407-co-poly (methacrylic acid) was designed to target 5-fluorouracil (5-FU) in colorectal cancer [37]. pH-dependent swelling and release (hydrogen swelled at pH 7.4 and released more drug at pH 1.2) were maintained in a controlled manner for a more extended period of 5-FU. The toxicity assessment on rabbits also showed the compatibility of this hydrogel with biological systems. Thus, this hydrogel formulation is promising to be used in drug delivery to the colon. 

**Figure 4:**
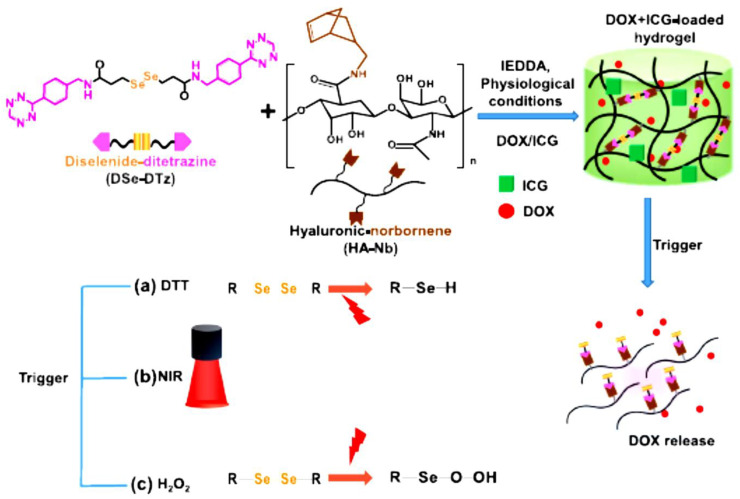
Illustrative synthesis of multi-responsive HA-derived hydrogel diselenide bonds cleavage mechanism under different triggers and the actions of drug release. Adapted from Jo et al. [38]. DTT: 1,4-dithiothreitol; NIR: Near-infrared.

In addition, multi-stimuli responsive HA-hydrogels were effectively formulated with cross-linker diselenide bonds for precise release of DOX as shown in Figure 4 [38]. DOX-loaded hydrogel resulted in an antitumor impact in breast cancer cells (BT-29). Incorporating indocyanine green (ICG) into the DOX-loaded hydrogel further improved the antitumor efficacy of DOX. Drug contents in DOX/ICG-loaded hydrogel were higher compared to DOX-loaded hydrogel, 94% and 4.54%, respectively. Thus, this study summarized that multi-responsive HA-hydrogel can be achieved by incorporating multiple cleavable bonds using diselenide bonds and the result showed a great efficacy of DOX-loaded hydrogel compared to free DOX and the addition of ICG has further improved the system.

Chemotherapeutic drugs such as DOX have inevitable drawbacks with poor targeting as well as poor clinical efficacy. Although CD44 is usually overexpressed in cancer cells, HA, known as CD44 receptor-mediated targeting, is a good choice of polymer to carry the anticancer drug more efficiently. In vitro studies showed a great efficacy of using HA as biomaterials, thus in vivo studies are demanding for future studies. A summary of studies on HA-based DDS is presented in Table 2.

### 2.3. Pectin

Pectin, a naturally occurring negatively charged polysaccharide is commonly found in plant cell walls of peaches, apples, and citruses. This anionic polysaccharide resembles HA and alginate, but it holds unique characteristics owing to its stiffening and solidifying abilities. These capabilities are vital in creating an excellent DDS for the gastrointestinal (GI) system. Notably, the excellent mucoadhesion and superior stability in the GI are due to its high resistance towards enzyme degradation (e.g., proteases and amylases) [39,40]. Pectin’s benefits to its gelling property make it a superior choice for oral insulin delivery compared to other anionic polysaccharides. However, pectin faces two major problems in the GI environment: the limited capacity of enterocytes to target the polymer and untimely drug release [39].

**Figure 5:**
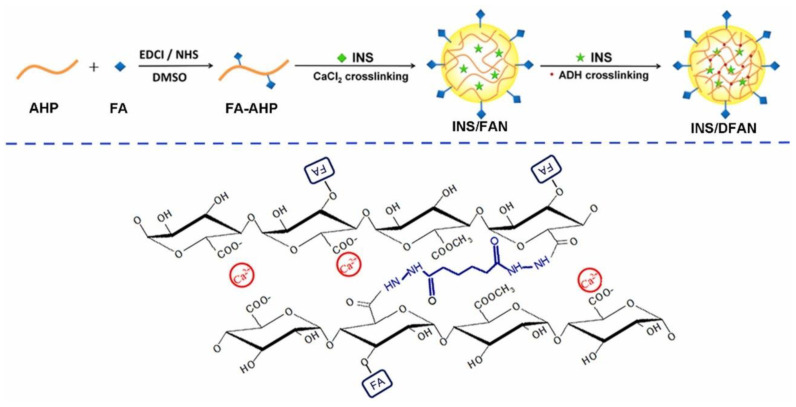
Synthesis structure of INS/DFAN dual-crosslinked. Adapted from Zhang et al. [39].

Therefore, Figure 5 shows a preparation of insulin DDS using folic acid (FA)-modified pectin nanoparticles (INS/DFAN). This system was prepared by a dual-crosslinking process using calcium ions and adipic dihydrazide (ADH) as crosslinkers to overcome the limitations [39]. Initially, pectin in this study was isolated from head residues of sunflower to produce low-methoxyl pectin (AHP). Findings from in vitro experiments showed the insulin dispersion behaviors of INS/DFAN influenced by the COOH/ADH molar ratio in the dual-crosslinking procedure. INS/DFAN can efficiently prevent the untimely release of insulin compared to ionic-crosslinked nanoparticles (INS/FAN). INS/DFAN also exhibited high encapsulation efficiency, excellent stability, and enhanced insulin delivery, whereas in vivo experiments on type 1 diabetic rats demonstrated improved hypoglycaemic effects and improved insulin bioavailability over INS/FAN. Overall, the combination of dual crosslinking and FA modification on pectin nano-vehicles was found to serve as a good strategy to enhance oral insulin delivery.

Next, a study by Bostanudin et al. proposed nanoformulations of amphipathically modified pectin-containing fusidic acid [41]. The amphipathic properties of a nanocarrier are important to surmount cell membrane impenetrability to boost drug permeation through the skin and to allow the delivery of both hydrophilic and hydrophobic particles or macromolecules into cells [41,42]. Amphipathically modified pectin (GBE-PEC) fabrication material is then converted into spherical nanostructures (NSs) [41]. Encapsulated fusidic acid was released in a more controlled manner (loading degree 14.9%), and in vitro interaction with HaCaT cells showed a non-cytotoxicity profile and demonstrated a greater (two-fold) penetration rate through the Strat-M^®^ membrane compared to the native pectin NSs. The improvement in the amphipathic properties drove the efficiency of poorly penetrating actives such as fusidic acid through percutaneous delivery. 

Pectin has been shown as a good polymer to carry drugs through the oral delivery system, influenced by its ability in gelling to produce high stability and high resistance towards the harsh environment in the GI. Integrating pectin polymer for oral drugs such as diabetic drugs represents a therapeutic window for diabetes treatments. The summary of studies on pectin-based DDS is presented in Table 3.

### 2.4. Starch

Starch is the amplest biopolymer obtained from plant sources; it is disposed of two macromolecules and linear- and branched-chain polymers. Its sensitivity to physical and chemical alterations and its capability to form thermoplastics are among its unique properties [43]. Starch is a major excipient in the pharmaceutical industry, especially in oral drug delivery. However, there are several major drawbacks of starch; 1. weak mechanical properties, 2. fast degradation in the body, 3. extreme viscosity after heating, 4. not soluble in cold water, and 5. capable of decomposing again [44]. Therefore, to overcome the limitations of this high-potential biomaterial, much current research was conducted with a new formulation strategy. 

Nanoparticles can serve as co-distributers of anti-inflammatory medicines and reactive oxygen species (ROS) scavengers for inflammatory bowel disease (IBD) therapy. Curcumin can be conjugated with hydroxyethyl starch (HES) and loaded with dexamethasone (DEX) to create DEX-loaded HES-CUR nanoparticles (DHC NPs) [45]. α-amylase is present in inflamed colon-degraded HES and allows the drugs to be released in an α-amylase-responsive way. DHC NPs also showed effective internalization and cytocompatibility with macrophages. DHC NPs are significantly greater in efficacy compared to free DEX in treated ulcerative colitis in the in vivo study. Thus, the results from this study stated that DHC NPs are having a therapeutic window for the emergence of novel oral formulations for IBD rehabilitation. Carvacrol is a phenolic compound that is prone to degrade in harsh conditions, especially in the GI. This drug requires a good carrier to protect them and ensure the optimal release of the drug in a controlled pattern. Thus, incorporating carvacrol in starch nanofiber by electrospinning a starch solution serves a great deal [46]. Carvacrol was delivered successfully by resisting in vitro digestion and produced a 50% decline in tumoral cells in glioma cells of C6 rats. Carvacrol-loaded starch nanofibers are safe and non-toxic to the cell. This suggests a good formulation for cancer treatment.

**Figure 6:**
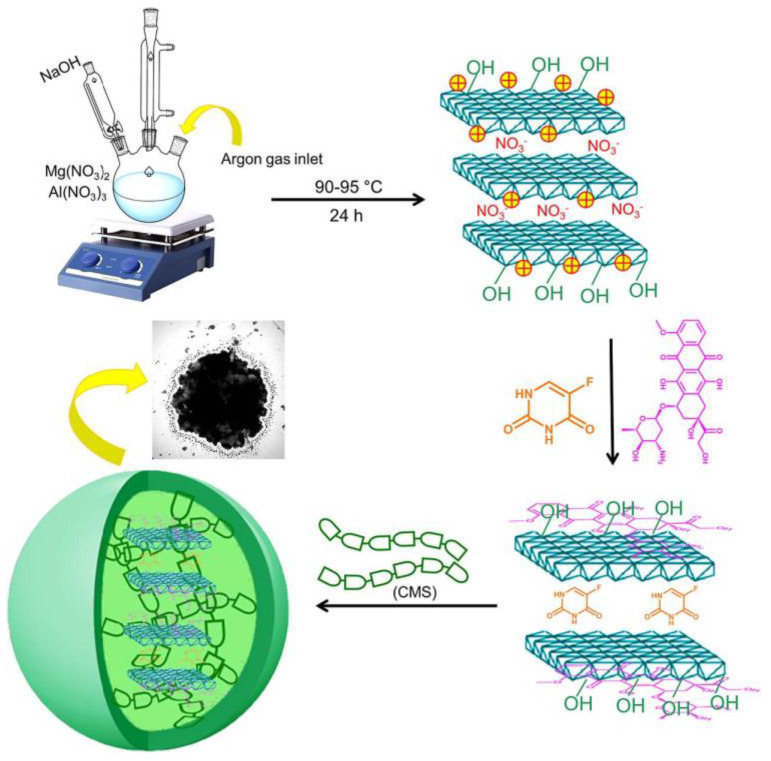
Graphic representation of the LDH(Mg–Al) production, co-drug payload, LDH(Mg-Al)@DOX,5-Fu coating with CMS, and the suggested method for the drug molecule releases from CMS@LDH(Mg–Al)@DOX,5-Fu microspheres. Adapted from Ranjbar et al. [44].

Targeting colon cancer therapy concurrently uses co-loaded DOX and 5-Fu on as-created layered double hydroxides LDH(Mg-Al) (LDH(MgAl)@DOX,5-Fu) [44]. The system was then encapsulated into carboxymethyl starch, forming CMS@LDH(MgAl)@DOX,5-Fu microspheres. It presented a reassuring constant drug release pattern and precise release profile of DOX and 5-Fu of ~22% and ~29%, respectively. The findings suggested the potential of the proposed microsphere for oral co-drug delivery. Figure 6 below illustrates the research of CMS@LDH(MgAl)@DOX,5-Fu microspheres.

In summary, colonic drug delivery requires a targeted system accounting for the harsh physiological state of the GI that has an acidic environment and a high concentration of hydrolytic enzyme. Therefore, proposing a good DDS is crucial for curing digestive system disorders: Crohn’s disease, ulcerative colitis, and colorectal cancer. Starch has proven itself as a promising natural polymer for DDSs in the GI through several successful studies on starch formulations. The summary of studies on pectin-based DDSs is presented in Table 4.

### 2.5. Xanthan Gum (XG)

XG is a natural polymer originating from the fermentation process of the microorganism Xanthomonas campestris [47]. Its high molecular weight is owed to its unique chemical properties; i.e., it is composed mainly of a β-1,4-D-glucopyranose glucan backbone with a pendant trisaccharide side chain, disposed of mannose (β-1,4) and glucuronic acid (β-1,2), as well as terminal mannose residues. This chemical structure makes XG polysaccharides with polyanionic characters. A more detailed chemical structure of XG was discussed [48]. XG stimulated the thickening behavior or assisted suspension in aqueous solutions by influencing the temperature and pH that affect the viscosity of XG. XG confers weak gel-like properties as an outcome of its 3D association with XG chains [49]; thus, hydrogel serves as a better medium for XG through crosslinked 3D networks of hydrophilic polymers through physical–chemical methods. Although XG hydrogel’s biocompatibility is well established [50], some disadvantages such as poor mechanical strength, harsh gelation conditions, and lack of cell attachment moieties are still something appealing to be explored for further improvement. Thus, subsequent studies discuss recent research on XG modification. 

XG has been widely used in incorporating diabetes medication, and the following studies are discussed. The repaglinide drug to treat type 2 diabetes was loaded into hydrogel particles of XG derivatives, carboxyethyl XG and carboxymethyl XG, in a ratio of 1:2 (i.e., maximum drug entrapment efficiency of 92%) [51]. The system released 97% of the drug in 4 h stimulated by GI pH and prolonged drug release for 8 h. The repaglinide’s amorphous dispersion was observed after the entrapment. The clinical benefit shown by this system was a reduction in blood glucose levels (maximum 52.8%), indicating that this system is beneficial in future diabetes treatment. Another diabetes drug is glibenclamide (i.e., an oral agent) loaded into mouth-dissolving films (MDFs), named GMDFs [52]. XG was added into the composition of GMDF as a film matrix through the solvent casting method, and the GMDF3 formulation composed of 200 mg presented the highest drug entrapment (96.1 ± 5.89). This GMDF also showed an instant release of the drug, rapid dissolution, and optimum mechanical strength. The GMDF1-3 showed a 96–98% discharge of the drug, and a 94% and 90% discharge of the drug in GMDF4 and GMDF5, respectively. These results suggested an XG film matrix can produce a stable DDS for glibenclamide in diabetes treatment using MDF. 

Another MDFs was created using XG by loading amlodipine (i.e., hypertensive drug) aiming to rapidly release the drug for the faster relief of hypertension [53]. This formulation shows exceptionally rapid results (i.e., complete drug release within 10 min) and drug release dispersion with optimum mechanical strength. Accelerated drug release in this formulation represented a therapeutic window in hypertension treatment. Hesperidin (HSP) (i.e., drug against *P. vulgaris*) was characterized as having low solubility. Therefore, Alam and colleagues produced HSP-enabled gold nanoparticles (AuNPs) stabilized with xanthan gum (XA), indicated as HSP@XA@AuNPs. HSP@XA@AuNPs gel was also prepared by integrating the formulation into a Carbopol gel base [54]. The results showed greater effectiveness in drug release by HSP@XA@AuNPs gel compared to HSP@XA@AuNPs, 86.26% and 73.08%, respectively. In addition, the gel demonstrated antimicrobial activity as opposed to *P. vulgaris* (i.e., minimum inhibitory concentration of 1.78 µg/mL). In conclusion, the HSP@XA@AuNPs gel may represent a new strategy to inhibit *P. vulgaris* infection.

**Figure 7:**
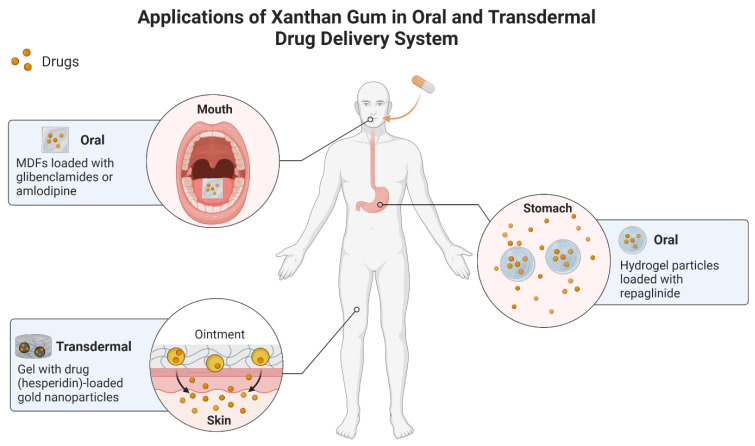
Summary of applications of xanthan gum (XG) in oral and transdermal drug delivery systems (DDS). Oral DDSs can be achieved by incorporating XG-based hydrogel particles loaded with repaglinide to treat type 2 diabetes and XG-based mouth-dissolving films (MDFs) loaded with glibenclamide to treat type 2 diabetes or amlodipine to treat hypertension. Transdermal DDSs can be achieved by incorporating XG-based gel with hesperidin-loaded gold nanoparticles to treat *P. vulgaris* infection.

In summary, XG’s limitation on its mechanical strength has been successfully defeated by XG derivative modification on its backbone through carboxymethylation and acetylation as shown by Patel et al. [51]. The steady hydrogel networks developed the subsequent formation of polymeric ionic bridges or synchronized ties among carboxylate ions of XG derivatives and aluminum ions. In addition, adding XG in formulations improved the DDSs of MDFs to a higher par [52,53]. Moreover, XG has been utilized in the oral and transdermal DDSs of various drugs. Figure 7 illustrates the summary of applications of XG in oral and transdermal DDSs, whereas the summary of studies on XG-based DDSs is presented in Table 5.

### 2.6. Dextran

Dextran is a naturally biodegradable polymer obtained from microbe sources. It is feasibly isolated from numerous Gram-positive, facultatively anaerobe cocci (e.g., leuconostoc and streptococcus strains) [55]. Dextran is highly soluble in water, DMSO glycerol, and ethylene glycol because of its neutral complex amylopectin-chain glucan composed of α-1, six glycosidic linkages in the middle of glucose monomers [56]. Dextran-based nanocarriers have great aqueous solubility, which can accelerate drug suspension. Dextran is also a non-toxic biopolymer as it does not accumulate toxicity in the GI compared to synthetic polymers due to its ability to metabolize with digestive enzymes, making dextran a favorable polymer in oral drug delivery [55]. However, natural dextran still retains shortcomings regarding its physiochemical properties such as surface-immobilized dextran limiting cell adhesion and spreading [57], which limits its utility for tissue engineering [58]. Thus, it requires the modification of its backbone through conjugation with drugs, amidation, carboxymethylation, acetylation, cross-linking, and grafting with other natural, synthetic, or semisynthetic polymers [59]. Dextran is supported by modification as shown in the following studies. 

Dextran is an example of an ultrasound-responsive polymeric material (i.e., combination of imaging techniques plus therapeutic) in DDSs that represents cost effectiveness and is non-invasive and more targeted compared to other internal or external stimuli-responsive (e.g., UV-, thermal, and pH-responsive) materials [60,61]. Dextran has been integrated into the novel nanotechnology of nanodroplets for ultrasound-induced cancer treatment [62]. Dextran stabilized perfluorohexane nanodroplets comprising the DOX drug. The outcomes are reported as follows: its particle size and encapsulation effectiveness were significantly amplified by elevating polymer concentrations, and in vitro analysis showed a biphasic drug release system of 82.95% of the DOX from the optimal formulation (0.1% *w*/*v* dextran, 24,000 rpm homogenization speed and 500 µg DOX content) after 10 min of exposure to ultrasound. Thus, this formulation showed a therapeutic window in ultrasound-induced cancer treatment. 

NIR light has intelligently worked with DDSs by having robust trigger levels, deeper dissemination through concerned sites, and a small number of side effects compared to UV light [63]. Topical photothermal hydrogel for NIR-controlled DDSs was prepared by the polymerization of vinyl-functionalized dextran (DexIEM), vinyl-modified graphene oxide (GM), and Laponite; the hydrogel was then inserted with ciprofloxacin (i.e., an antibacterial drug) [64]. Ciprofloxacin in the DexIEM-GM-Laponite hydrogel dispersion remained in a NIR-controlled manner in an ex vivo trial. Interestingly, this hydrogel system exhibited excellent performance in terms of antibacterial effects and good compatibility with blood. This study suggested a novel system for a NIR-responsive DDS. Celastrol (Cel), rheumatoid arthritis (RA) drug, was loaded into nanoparticles made up of modified dextran (dextran-sulfate-PVGLIG), named DPC [65]. It resulted in a high entrapment of DPC@Cel micelles (around 44.04%) and a zeta potential of −11.91 mV. The nanoparticles effectively delivered the drug to the inflammatory joint and metalloproteinase-2 (MMP-2) at the accelerated Cel released through in vitro observation. An in vivo trial confirmed that DPC@Cel improved anti-RA effects and decreased systemic toxicity in comparison to free Cel. This indicated an effective system of Cel delivery to the target site. 

Lastly, a pH-sensitive dextran-based micelle scheme was fabricated using an ester click reaction of copper-free azide-propiolate, self-constructed from amphiphilic dextran-graft-poly(2-(diisopropylamino) ethyl methacrylate-co-2-(2′,3′,5′-triiodobenzoyl) ethyl methacrylate), or dextran-g-P(DPA-co-TIBMA) [66]. DOX-loaded dextran-g-P(DPA-co-TIBMA) micelles showed a reduced speed release of DOX at pH 7.4 but were significantly sped up under an acidic state (pH 6 and 5). Micelles of dextran-g-P(DPA-co-TIBMA) optimally released DOX into MCF-7 cells. DOX-loaded dextran-g-P(DPA-co-TIBMA) was found to have excellent anticancer efficacy and effectively reduced the growth of tumors with little body weight reduction in in vitro and in vivo studies, respectively. Both in vitro and in vivo studies demonstrated a promising strategy using this system in tumor suppression. 

**Figure 8:**
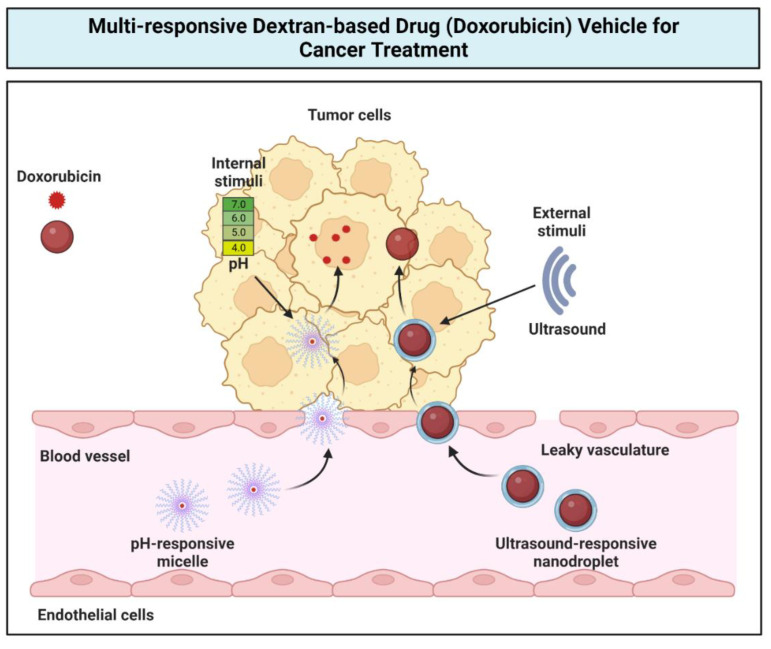
Multi-responsive dextran-based drug delivery vehicle to deliver doxorubicin (DOX) drug for cancer treatments. DOX is loaded into dextran-based micelles that are responsive to internal stimuli (i.e., changes in the pH tumor environment). DOX also can be loaded into a dextran-based nanodroplet that is responsive to external stimuli such as ultrasound. This multi-responsive DDS allows drugs to be delivered in a more targeted manner such as in tumor cells. The micelles and nanodroplets enter the tumor environment through leaky vasculature. DOX will only be released into the tumor cells upon response to pH changes or ultrasound stimuli.

To conclude, the recent applications of dextran polymeric materials have been directed towards multi-stimuli responsive DDSs such as pH, NIR light, and ultrasound. Stimuli-responsive materials are of vast importance because of their capacity to undergo adjustment of their properties in response to their environment. For example, the pH-responsive polymer is characterized by its features of moieties that can donate or accept cations upon an environmental change in pH [67]. However, light-reactive polymers use light as a versatile stimulus through their subsequent light-responsive moieties which can be related to photoinduced isomerization and/or photochromism [68]. This smart polymer produces more refined applications, due to the variability that is introduced to the responsiveness. Figure 8 presents the multi-responsive dextran-based drug (DOX) vehicle for cancer treatments. The summary of studies on dextran-based DDSs is presented in Table 6.

## 3. Applications of Natural Polymeric Fe_3_O_4_ MNPs Composites in DDSs

**Figure 9:**
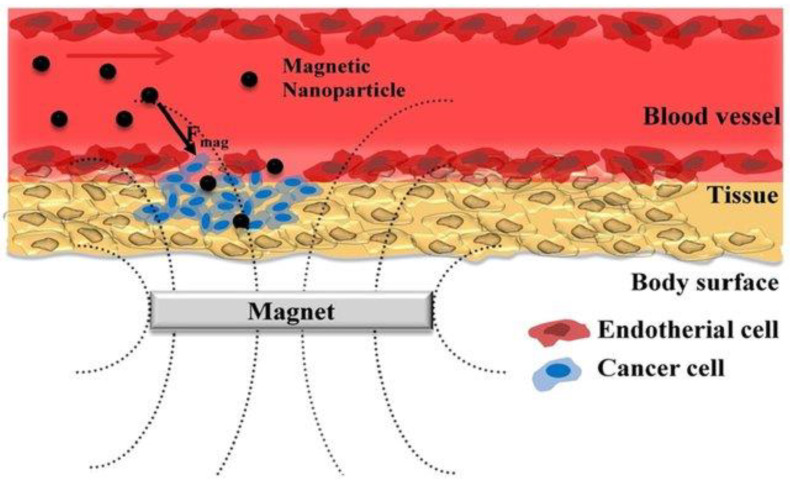
Schematic of an EMF-influenced magnetic drug delivery system. Adapted from Park et al. [71].

The three main goals of drug delivery are to deliver medicine to the right location, reduce the medication’s adverse effects on neighboring organs or tissues, and control the medication’s release to stop the cycle of over- and under-dosing [69,70]. These objectives may be accomplished by utilizing Fe_3_O_4_ MNPs because of their distinctive characteristics, magnetism, and simplicity of manipulation using an EMF, which sends drug-carrying Fe_3_O_4_ MNPs to the targeted location directly (as shown in Figure 9). 

Interestingly, Fe_3_O_4_ MNPs can be used to control the release of drugs or other active agents in drug delivery applications. The use of Fe_3_O_4_ MNPs in controlled release systems is based on the ability of magnetic fields to exert forces on the MNPs and the associated drugs. When exposed to an EMF, Fe_3_O_4_ MNPs align themselves along the field lines, creating a magnetic gradient force. The drugs can be adsorbed or encapsulated onto or within the MNPs, and the combined system can be used to control the release of the drugs. The effect of Fe_3_O_4_ MNPs on controlled releases can be influenced by several factors such as the size and morphology or anisotropy of the Fe_3_O_4_ MNPs, and the strength and duration of the applied magnetic field. By tailoring these factors, it is possible to achieve a wide range of release profiles.

### 3.1. Effect of Fe_3_O_4_ MNPs Size in DDSs

The size of Fe_3_O_4_ MNPs can have a significant influence on their ability to assist in DDS. The fine size of Fe_3_O_4_ MNPs contributes to the large surface-to-volume ratio, which allows for the greater loading of drugs [72]. Then, the loaded drug molecules on Fe_3_O_4_ MNPs can be delivered into the body and concentrated in a local area (avoiding damage to other tissues) via the effect of EMF. Shapiro [73] demonstrated that a series of EMFs may force a magnetic carrier through a central area, creating a focus at a deep target. The Fe_3_O_4_ MNPs may also be heated in a magnetic field (MF) to cause the release of a drug. This can result in a higher drug payload and improved efficacy of the drug delivery system [74,75]. Moreover, the size of MNPs may affect their magnetic properties. Smaller Fe_3_O_4_ MNPs have a higher magnetic moment per unit volume, which makes them more responsive to EMFs. This can be used to control the release of drugs in a targeted way. Despite that, the tiny size of Fe_3_O_4_ MNPs is a constraint as it causes magnetic agglomeration when the EMF is withdrawn. Once the EMF is withdrawn, it is challenging to maintain the Fe_3_O_4_ MNPs in the targeted organ, which causes them to agglomerate. Additionally, it is challenging to aim particles, maintain them near the target, and endure the drag of blood flow when the size is tiny as it suggests a magnetic response of diminished strength [76]. Targeting is presumably more effective in regions with slower blood flow, particularly when the EMF is close. 

On the other hand, larger MNPs can be more stable and less prone to aggregation, which can increase the circulation time in the body and improve the targeting of the MNPs to specific regions of the body. However, larger MNPs have a lower surface area-to-volume ratio, subsequently limiting the amount of drugs that can be loaded onto them. Therefore, it is necessary to regulate nanoparticle sizes throughout the preparation phase in order to efficiently use them as medication carriers. 

Therefore, these problems might be addressed by combining Fe_3_O_4_ MNPs with the appropriate polymers. It provides the particles with long-term stability by adding a layer of protection that encourages repulsive forces to counterbalance the magnetic and van der Waals forces on the magnetic particles [77]. Moreover, Schneider-Futschik and Reyes-Ortega [78] stated that the safest Fe_3_O_4_ MNPs and the coating are formed by a natural polymer.

### 3.2. Effect of Morphology or Anisotropy of Fe_3_O_4_ MNPs in DDSs

The morphology or anisotropy of Fe_3_O_4_ MNPs significantly influence their magnetic response. Anisotropy refers to the directional dependence of the magnetic properties of a material. MNPs can have either isotropic or anisotropic properties, depending on the method of synthesis and the shape of the particles. Anisotropic Fe_3_O_4_ MNPs have a preferred direction of magnetization, which can be influenced by the shape of the particles. For example, rod-shaped Fe_3_O_4_ MNPs have a higher magnetic anisotropy than spherical MNPs and tend to align along the long axis of the particle when exposed to an EMF. In addition, compared to spherical micelles, filomicelles have shown a larger potential for encapsulating anticancer drugs and more apoptotic efficacy. Chen et al. [79] investigated in vivo the anticancer effects of different micelle shapes and revealed that filamentous micelles had the best DOX loading and encapsulation capabilities. Other investigated shapes of nanoparticles reported were rod, worm, and bead. According to the literature, rod-shaped and non-spherical nanoparticles have a longer blood circulation duration than spherical nanoparticles [80,81]. This may be due to macrophages that are less active in phagocytic activity when exposed to rod-shaped particles than when exposed to spherical ones [82]. On the other hand, spherical nanoparticles may provide an even surface coating and conjugation of ligands in surface modification; thus, more drugs can be conjugated on the surface of nanoparticles for improved drug release at the targeted spot and therefore display larger cellular toxicity [83].

In addition, the morphology of Fe_3_O_4_ MNPs can influence their magnetic properties. For example, Fe_3_O_4_ MNPs that have a core–shell structure have different magnetic properties than solid Fe_3_O_4_ NPs. The shell material can also have a significant impact on the magnetic properties, as it can provide a protective barrier for the core and prevent oxidation or degradation. In a recent study of a micelle system by chitosan-based Fe_3_O_4_ MNPs undergoing surface modification by a shell of mesoporous silica and *γ*-glycidoxypropyltrimethoxysilane using a silane coupling method to study the drug release behavior of DOX, the observation resulted in faster drug release at pH 6.0 than at pH 7.4 [84]. Other studies of pH-responsive Fe_3_O_4_ MNPs implemented surface functionalization alteration by incorporating UiO-66-NH_2_ (i.e., zirconium-based functional organic metal framework) [85]. Moreover, double core–shell MNPs of Fe_3_O_4_@SiO_2_@Tann showed the co-delivery of DOX and methotrexate (i.e., anticancer drugs) was significantly higher in pH 5 than pH 7.4 [86]. 

Overall, the magnetic response of the Fe_3_O_4_ MNPs is highly dependent on their morphology and anisotropy. The ability to control these parameters is important for their application in drug delivery applications.

## 4. Applications of Polymeric Fe_3_O_4_ MNP Composites in DDSs

MNPs offer a great interest in a wide range of research including DDSs. Each MNP performs in a single magnetic domain and possesses superparamagnetic behavior, as shown by Fe_3_O_4_ MNPs. Superparamagnetic NPs have a low risk of forming agglomerates at room temperature through two special behaviors: 1. huge constant magnetic moment, 2. enormous paramagnetic atom with a rapid response to applied magnetic fields with negligible remanence and coercivity, which make them favorable MNPs in DDSs [87]. However, naked metallic NPs are highly active chemical components which can also easily oxidize in air and cause magnetism loss and dispersibility. Thus, various studies have been conducted to coat MNPs with natural polymers as discussed in this section. Of note, protection shells based on natural polymers not only stabilize the MNPs but can undergo further functionalization of their surfaces depending on the desired application. Fe_3_O_4_ MNPs can be guided to deliver drugs to the target site by localized EMFs. The magnetic particles should render high-saturation magnetization to control the movement of particles in the blood by moderate EMFs and to accumulate the particles close to target sites [88]. For example, high saturation magnetization has also been proposed to deliver drugs for breast cancer therapy in a more efficient way [89] and to enhance the biocompatibility of MNPs [90]. Table 7 shows the recent development of natural polymeric Fe_3_O_4_ MNP composites in DDSs. 

**Figure 10:**
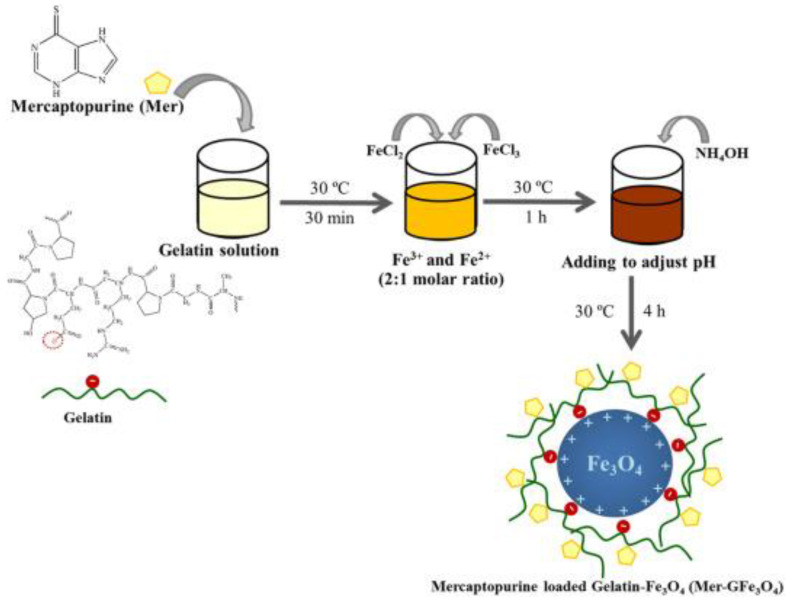
Proposed mechanism interaction between Fe_3_O_4_, gelatin, and loaded drug. Adapted from Sirivat et al. [91].

**Figure 11:**
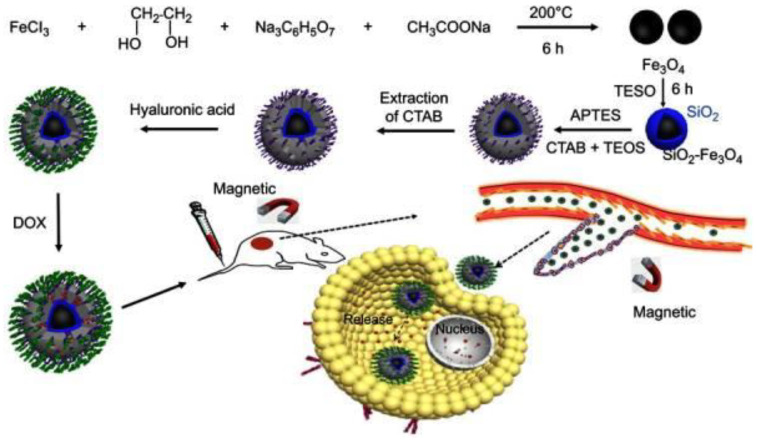
A proposed creation of HA-MSNs for targeted cancer treatment in vivo and pH-responsive drug release following specific binding with cancer cells. Adapted from Fang et al. [95].

Sirivat and Paradee [91] prepared gelatin-coated Fe_3_O_4_ (GFe_3_O_4_) composites, as illustrated in Figure 10, with superparamagnetic characteristics that are significant in preventing particle agglomeration and facilitating the rapid dispersion of the particles upon the removal of the EMF [92]. Moreover, although the use of HA-drug conjugates in DDSs has received a lot of attention in recent years [93,94], they do not effectively bind to targeted cancer cells. Fang et al. [95] overcame this issue by developing a more efficient drug delivery system, as displayed in Figure 11 through the modification of mesoporous silica-coated Fe_3_O_4_ nanoparticles (MSNs) with HA, then loading with DOX. The DOX-HA-MSNs showed remarkable targeting ability as well as efficient anticancer activity both in vitro and in vivo.

**Figure 12:**
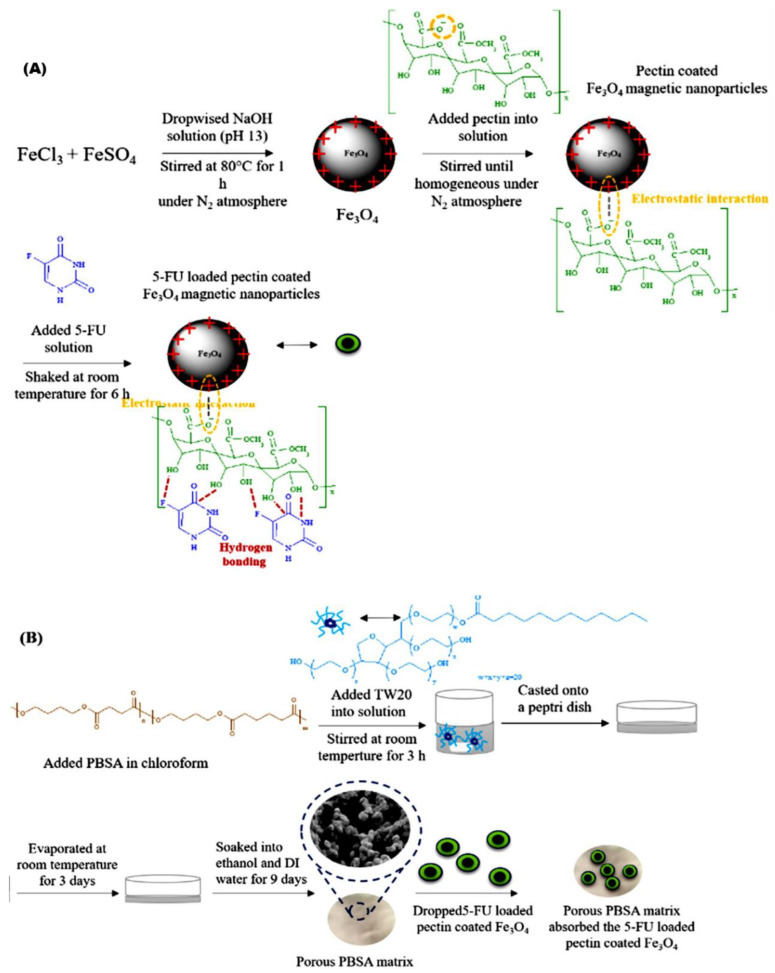
Illustration of (**A**) 5-fluorouracil-loaded pectin-coated Fe_3_O_4_ MNPs; (**B**) porous poly(butylene succinate co adipate). Adapted from Viratchaiboott et al. [96].

**Figure 13:**
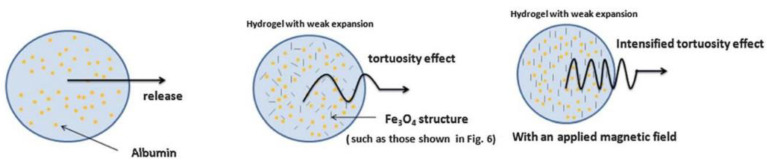
Albumin release from a hydrogel without and with an applied magnetic field: a proposed mechanism. Adapted from Guilherme et al. [97].

Viratchaiboott et al. [96] suggested that the mechanism of Fe_3_O_4_ coated with pectin was through electrostatic attraction (as illustrated in Figure 12). Then, the 5-FU was loaded onto the Fe_3_O_4_/Pectin through hydrogen bonding. The results found that the duration of the drug release was shortened while the drug diffusion coefficient increased under MFs, which resulted from the magnetic attraction between the magnets and the MNPs. In addition, Figure 13 shows that Guilherme et al. [97] investigated the albumin release mechanism from the starch polymer with and without Fe_3_O_4_. According to the authors, the albumin release mechanism of the starch without Fe_3_O_4_ was controlled via macromolecular relaxation. The albumin release for starch with Fe_3_O_4_ was more reliant on anomalous transportation when an EMF was applied, which amplified the tortuosity effect. As they offered a greater sustention of the drug than the in vitro release experiment, the starch/Fe_3_O_4_ is more suitable for application as an oral drug delivery system.

Similarly, Bueno et al. [98] also reported novel antimicrobial materials based on XG, Fe_3_O_4_ MNPs, and bovine serum albumin (BSA) that were designed to promote the release of amoxicillin to the medium. The authors highlighted that the release of amoxicillin from the patches was successfully stimulated by applying an EMF. Another example from microbe sources is Dextran, coated with Fe_3_O_4_ MNPs and loaded with CUR for the treatment of lung cancer [99]. The observation showed that owing to curcumin’s poor solubility, the drug release is greater in CUR-Dextran-Fe_3_O_4_ nanoparticles and slower in curcumin solutions. Overall, the use of EMFs to direct polymeric Fe_3_O_4_ MNPs composites to a particular location demonstrates the interesting possibilities of DDSs. 

Based on the abovementioned studies on DDSs using Fe_3_O_4_ MNPs, there are two suggested routes of administration discussed, which are the oral and transdermal routes of administration for drugs loaded with MNPs. Oral route administration is considered common in delivering drugs using nanotechnologies. This is due to high patient acceptance, convenience, lack of pain, and easy monitoring. The unstable environment of the GI with its acidic pH and various enzymatic activities leads to the degradation of proteins, thereby reducing the therapeutic values of the drugs. Reconsidering this limitation on oral route administration, recently, there have been several studies incorporating the benefits of natural polymers and Fe_3_O_4_ MNPs such as oral drug delivery for colon cancer treatment [100,101]. The pH-sensitive nanocomposites for oral drug delivery stabilize the release of drugs to a specific area, such as the colorectal area monitored by an external magnetic source. Thus, this prevents the release of drugs in the stomach, which may cause serious side effects on the digestive system. 

**Figure 14:**
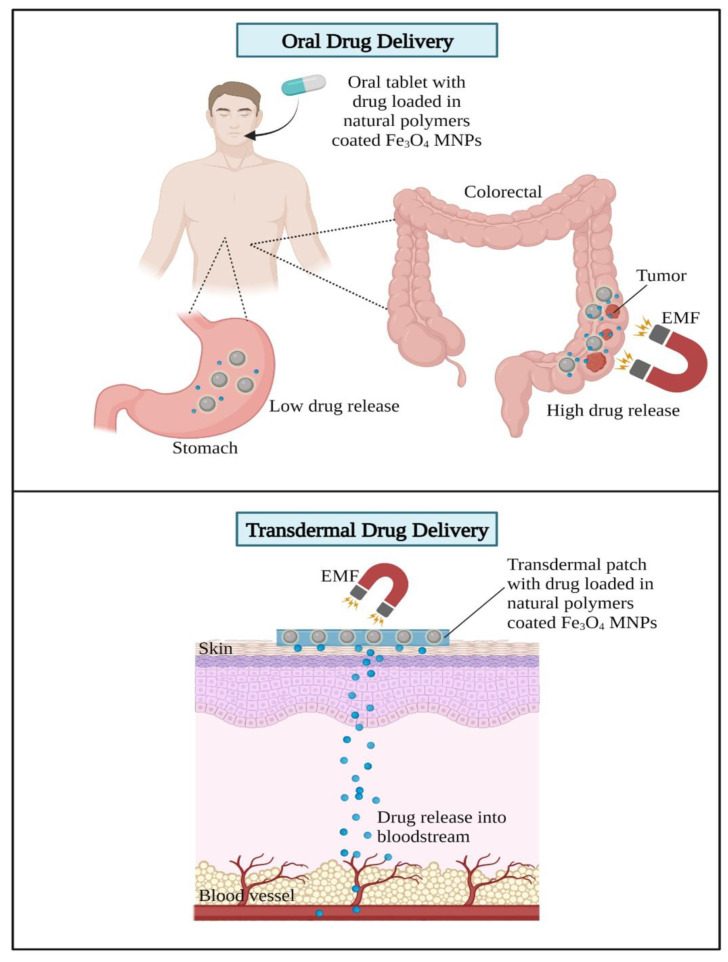
Mechanism of actions of oral drug and transdermal drug delivery by Fe_3_O_4_ MNPs. Oral drug delivery: Oral route administration is achieved by ingesting an oral tablet containing a drug loaded in the natural polymer coated with Fe_3_O_4_ MNPs. Stable Fe_3_O_4_ MNPs only produce a slow drug release in the stomach but high drug release in the targeted site such as in the colorectal region upon exposure to an external magnetic field (EMF). Transdermal drug delivery: a transdermal patch with the drug loaded in natural polymers coated with Fe_3_O_4_ MNPs is placed on the skin, and an EMF is applied to trigger drug release from the Fe_3_O_4_ MNPs directly into the bloodstream.

Next, the transdermal administration of drugs by delivering them into the bloodstream through the skin using a patch has been recently proposed in Fe_3_O_4_ MNPs [96]. The transdermal administration of drugs prevents the first-pass metabolism effects of drugs, which are able to lower the dose of drugs administered and reduce toxicity [102]. Harnessing the advantages of MNPs under the combined influence of magnetic and electric fields increases the efficacy of transdermal drug delivery and allows for a more controlled release of the drug at the target site. The mechanism of action of Fe_3_O_4_ MNPs in delivering drugs using oral and transdermal routes of administration is illustrated in Figure 14.

Nevertheless, the consideration of the interaction between an MF and living cells is crucial in the applications of Fe_3_O_4_ MNPs. This is because biomagnetic effects have been widely shown to disrupt normal physiology at the cellular or organism levels [103,104,105]. Controlling the levels of EMFs exerted during delivering drugs in MNPs must be carefully monitored, and alternating MFs with a frequency between 100 and 500 kHz is considered a safe frequency for humans [106]. High and ultrahigh MFs exert biological effects such as the long-term impairment of the vestibular system in mice [107], and MFs change the orientation and morphology of mitotic spindles in human cells [108]. Thus, stable Fe_3_O_4_ MNPs by incorporating natural polymers have been widely produced to minimize the levels of EMFs required in magnetic drug targeting (MDT) and simultaneously hinder the negative effects of MFs on living cells.

**Table 7:** Recent studies on natural polymeric MNP composites in DDSs.

Gelatin-Based Fe_3_O_4_ Composites						
Formulation	Drug Delivery Vehicle	Platform	Treatment	Loaded Drug	Observation	Ref.
DG/FA NPs	Nanoparticles	In vitro	Breast cancer	DOX	Decreased 48% cell viability.	[109]
Gel-MNPs	Nanocomposite	N/A	Lung and breast cancer	CUR	Loaded drugs are released in a pH-dependent manner, with a greater release rate in a moderately acidic environment.	[110]
Fe_3_O_4_/GQDs@GM	Microspheres	In vitro	Breast cancer	CUR	Exhibited high CUR loading capacity.	[111]
Alg-Gel/Fe_3_O_4_	Hydrogel	In vitro and In vivo	Hela	DOX	Exhibited higher drug release value in acidic conditions.	[112]
**HA-based Fe_3_O_4_ composites**						
CDHA–MGO	Nanocomposite	In vitro	Tumor	DOX	Targeting CD44 to accumulate inside tumor cells via HA conjugation.	[113]
Fe_3_O_4_-HA	Nanoparticles	In vitro and In vivo	Tumor	DOX	Enhanced antitumor and anti-metastasis effect.	[114]
Fe_3_O_4_@HA NPs	Nanoparticles	In vitro	Breast cancer	DOX	Enhanced DOX cancer-targeting capabilities.	[115]
MGO@CD-CA-HA	Nanocomposite	In vitro and In vivo	Liver cancer	CPT	Significantly reduced tumor growth (more than 90%).	[116]
PC/HA@DOX-Fe_3_O_4_	Nanoparticles	In vivo	Xenograft tumor	DOX	Enhanced tumor growth suppression efficacy and significant DOX tumor-targeting capabilities.	[117]
**Pectin-based Fe_3_O_4_ composites**						
Pectin/Fe_3_O_4_	Nanoparticles	In vitro	Skin	5-FU	Higher diffusion coefficients and shorter duration periods increased 5-FU release.	[96]
Fe_3_O_4_/Pectin	Nanoparticles	In vitro	Colorectal	BHT	Best antioxidant activities against DPPH.	[118]
AP-MA/PNIPAAm/Fe_3_O_4_	Microgels	SGF and SIF	Colon cancer	CUR	Under the impact of EMF, a gradual and sustainable CUR release was made.	[100]
PEC-GO-Fe_3_O_4_	Nanocomposite	In vitro	*Aedes aegypti* larvae	Permethrin	Enhanced drug loading and release performance up to 16 h.	[119]
Pec-gPolyDMAEMA@Fe_3_O_4_	Nanoparticles	In vitro	Tumor	5-FU	A 50 mT magnetic field dramatically (100%) boosted the 5-FU.	[120]
**Starch-based Fe_3_O_4_ composites**						
CMC/PAA/St-Fe_3_O_4_	Nanocomposite	In vitro	Colon cancer	5-FU	5-FU delivery to the intestinal fluid using an external magnetic source.	[101]
Fe_3_O_4_@CS-Starch/Cu	Nanocomposite	n.a.	Ovarian cancer	BHT	Ovarian cell viability decreased in a dose-dependent manner.	[121]
Fe_3_O_4_@Gr-IA/St-Alg	Hydrogel	In vitro	Wound healing	GFN	High efficiently loaded GFN and drug released in a controlled manner.	[122]
**Dextran-based Fe_3_O_4_ composites**						
Dextran@Fe_3_O_4_	Nanoparticles	In vitro	Prostate cancer	AUR	AUR release was considerably increased in an acidic medium.	[123]
Dextran-coated Fe_3_O_4_	Nanoparticles	In vitro	Lung cancer	DOX	Enhanced the drug concentration in the tumor cell by applying EMF.	[124]
Dextran/Fe_3_O_4_	Nanoparticles	In vitro and in vivo	Tumor	PBS	The dextran/Fe_3_O_4_ injection-induced tumors entirely vanished after 28 days.	[125]
Magnetic dextran	Microgel	In vitro	Tumor	DOX	DOX release profile that is both magnetic field and pH sensitive.	[126]

CUR: curcumin; N/A: not available; CPT: camptothecin; PTX- paclitaxel; BHT: butylated hydroxytoluene; GFN: guaifenesin; AUR: auraptene; PBS: phosphate-buffered saline; Ref: references.

### Biomedical Applications of Fe_3_O_4_ MNPs

Apart from Fe_3_O_4_ MNPs involvement as a drug delivery carrier, Fe_3_O_4_ MNPs have been widely used and commercialized in other biomedical applications. Firstly, MDT, as proposed by the above-mentioned novel studies, shows clinical efficacy and safety, thus warranting further studies and clinical trial enforcement. To date, there are currently no approved Fe_3_O_4_ MNPs for MDT. Several clinical trials were conducted on Fe_3_O_4_ MNPs for MDT. In 1996, the first phase I clinical trial was conducted in cancer patients to assess the efficacy of Fe_3_O_4_/anhydroglucose (ferrofluids)-loaded epirubicin [127] followed by phase I/II to assess the efficacy of DOX loaded in metallic iron-activated carbon [128] and metallic iron-activated carbon [129] in hepatocellular carcinoma patients. However, none of them presented with clear clinical success.

Moreover, Fe_3_O_4_ MNPs have been widely used as contrast agents in magnetic resonance imaging (MRI) to produce high-quality MRI. There are several FDA-approved Fe_3_O_4_ MNP contrast agents such as dextran-coated Fe_3_O_4_ MNPs (ferumoxide). The clinical trial result on ferumoxide (Resovist) showed enhanced MRI by significantly increasing the tumor/liver contrast [130] to 95% sensitivity in the detection of metastatic tumors [131] and to 27% high MRI quality using ferumoxides compared to non-enhanced images [132]. Moreover, ferumoxtran, with dextran-coated Fe_3_O_4_ MNPs, has also been approved through significant changes in the MR signal intensity of the blood pool and well-perfused organs (liver and spleen) that were noted on both nuclear magnetization T1- and T2-weighted images in a clinical trial [133]. Next, carboxydextran-coated Fe_3_O_4_ MNPs with ferucarbotran (VivoTrax) has recently shown efficacy in a multicenter clinical trial on sentinel lymph nodes (SLN) biopsy involving 220 breast cancer patients where ferucarbotran exceeded the threshold identification of SLN of 94.8% [134]. An exploratory clinical study has been conducted to lower the dose of ferucarbotran, and the results showed that a 0.5 ml dose was sufficient for SLN identification [135].

Harnessing the capability of Fe_3_O_4_ MNPs in immunomagnetic separation (IMS) techniques has led to the discovery of various biomarkers in biomedical research. One of the FDA-approved Fe_3_O_4_ MNPs for IMS techniques is the CELLSEARCH system from Janssen Diagnostics Inc. for metastatic breast cancer, metastatic colorectal cancer, and prostate cancer [136]. Another FDA-approved IMS system is the CliniMACS CD34 Reagent System by Miltenyi Biotec utilizing the anti-CD34 for clinical allogeneic stem cell transplantation in patients with acute myeloid leukemia [137]. Lastly, magnetic fluid hyperthermia (MHF) using Fe_3_O_4_ MNPs is a promising approach to cancer therapy [138] by producing sufficient heat to kill cancer cells. In 2018, the FDA approval of MHF NanoTherm therapy built by MagForce was accelerated after a clinical trial with prostate cancer patients [137].

## 5. Conclusions

Numerous studies have shown that Fe_3_O_4_ MNPs have strong magnetic characteristics that make them ideal for targeted medication delivery systems. However, Fe_3_O_4_ MNPs have the propensity to aggregate and cause toxicity in living organisms. Therefore, applying a polymer to their surfaces to increase biocompatibility and blood half-lives appears to have potential. It can be summarized that using natural polymers on Fe_3_O_4_ has gradually reduced their propensity to aggregate while simultaneously improving their biocompatibility and reducing their potential toxicity. On the other hand, these advantageous characteristics can be compromised by larger particle sizes and diminished magnetic properties. 

Despite the fact that natural polymeric Fe_3_O_4_ MNPs composites have several benefits for drug delivery applications, there are still significant difficulties with clinical applications. The efficacy of naturally polymeric Fe_3_O_4_ MNPs in vivo is currently unsatisfactory due to the fact that the majority of drug-delivery systems are examined in vitro. Therefore, in vivo testing should be progressively included in future studies. Additionally, in vivo conditions will be very difficult for natural polymeric Fe_3_O_4_ MNPs with weak physicochemical qualities. For example, natural polymeric Fe_3_O_4_ MNPs with poor magnetism characteristics in vivo find it hard to respond to EMFs. However, inappropriately boosting the amount of Fe_3_O_4_ MNPs in the polymer matrix might be hazardous to human health and compromise other qualities of intelligent responses, such as pH sensitivity. To enhance their applicability in the challenging physiological environment in vivo, more natural polymeric Fe_3_O_4_ MNPs should be designed with a sensible structure and chemical composition. More critically, additional research is needed to generate natural polymeric Fe_3_O_4_ MNPs with improved safety.

Moreover, we anticipate that the dual- and triple-response Fe_3_O_4_ MNPs based on natural polymers for drug delivery will play a more important role in the field of material application in the future. Additionally, biomimetic nanotechnology has recently attracted attention with its notion of wrapping polymeric nanoparticles (NPs) with cell membranes; for example, they were extracted from the cancer cell membranes. This has resulted in multi-immune responses to different tumor antigens attributable to receptor–ligand interactions in surface cells, thereby enhancing biological adhesion and immune clearance. The building of innovative structures that have the capability of mimicking the functionalities of biological components in order to maximize biointerfacing and biological cue manipulation. This biomimetic concept can be adopted and further improved by using cancer cell membrane-based wrapped Fe_3_O_4_ magnetic nanoparticles with the polymers for cancer vaccine design and therapy development.

## Figures and Tables

**Table 1:** Summary of studies on gelatin-based DDS.

Gelatin-Based DDS						
Formulation	Drug Delivery Vehicle	Platform	Disease	Loaded Drug	Observed Effects	Ref.
Gelatin	Hydrogel-based MN	In vitro	Keloid scarring	Gallic acid QAGN	Produced a controlled drug release of QAGN. Downregulated the gene expression of fibroblasts.	[22]
Gelatin methacryloyl and β-cyclodextrin	Hydrogel-based MN	In vitro and in vivo	Melanoma cancer	Curcumin	Higher therapeutic efficacy compared to non-transdermal patches. Verified biocompatibility and degradability.	[25]
Gelatin and hydroxyapatite	NPs	In vitro	Lung cancer	Curcumin	Sustained release of curcumin. Higher increase in cellular internalization and toxicity towards A549 cells than free curcumin.	[26]
pH-sensitive gelatin	Microsphere	In vitro	Respiratory disease	Carvedilol	Rapid drug release under acidic state (pH = 1.2) and non-toxicity against Caco-2 cells	[27]

DDS: drug delivery system; MN: microneedle; NPs: nanoparticles; QAGN: quercetin-loaded amphiphilic gelatin nanoparticles; Ref: References.

**Table 2:** Summary of studies on HA-based DDS.

Hyaluronic Acid (HA)-Based DDS						
Formulation	Drug Delivery Vehicle	Platform	Disease	Loaded Drug	Observed Effects	Ref.
HA-tetraphenyl ethylene	Micelles	In vitro	Cancer	DOX	Great efficacy in unloading DOX through fast glutathione-triggered dissociation.	[33]
HA-modified	Halloysite nanotube	In vitro	Cancer	DOX	Enhanced the therapeutic efficacy of DOX. High antitumor efficacy in CD44-positive Hela cells.	[34]
HA–human serum albumin	Micelle-like NPs	In vitro and in vivo	Breast cancer	DOX	Greater cytotoxicity of MDA-MB231 cells. CD44-mediated internalization of nanoparticles.	[35]
HA	MN	In vivo	Alopecia	MDX	Enhancement of HDP cells. Reduced hair loss in alopecia.	[36]
HA	Hydrogel	In vitro and in vivo	Colorectal cancer	5-Fu	5-FU is retained in a coordinated manner for a more extended period. Toxicity assessment on rabbits also showed compatibility.	[37]
HA	Multi-stimuli responsive hydrogel	In vitro	Cancer	DOX	Antitumor effect in breast cancer cells (BT-29)	[38]

5-Fu: 5-fluorouracil; DDS: drug delivery system; DOX: doxorubicin; HA: hyaluronic acid; MN: microneedle; NPs: nanoparticles; Ref: references.

**Table 3:** Summary of studies on pectin-based DDS.

Pectin-Based DDS						
Formulation	Drug Delivery Vehicle	Platform	Disease	Loaded Drug	Observed Effects	Ref.
Folic acid-modified pectin	NPs	In vitro and in vivo	Type 1 diabetes	Insulin	Prevent the premature release of insulin. High encapsulation efficiency. Excellent stability. Enhanced insulin delivery. Improved hypoglycaemic effects on type 1 diabetes rats.	[39]
Amphipathically modified pectin	Spherical nano-structures	In vitro	Skin-related disease	Fusidic acid	Fusidic acid was released in a more controlled manner. HaCaT cells showed a non-cytotoxicity profile. Two-fold greater penetration rate.	[41]

DDS: drug delivery system; NPs: nanoparticles; Ref: references.

**Table 4:** Summary of studies on starch-based DDSs.

Starch-Based DDS						
Formulation	Drug Delivery Vehicle	Platform	Disease	Loaded Drug	Observed Effects	Ref.
Hydroxyethyl starch	NPs	In vitro and in vivo	Ulcerative colitis	Curcumin and DEX	Drugs released in an α-amylase-responsive manner. Effective internalization and cytocompatibility with macrophages. Greater in efficacy compared to free DEX.	[45]
Starch	Nanofiber	In vitro and in vivo	Cancer	Carvacrol	The system is resisting in vitro digestion. 50% reduction in cancer cells of rat C6 glioma cells.	[46]
Carboxymethyl starch -CMS@LDH(MgAl)@DOX,5-Fu	Microspheres	In vitro	Colon cancer	5-Fu and DOX	Sustained drug release pattern and controlled release profile of DOX and 5-Fu.	[44]

5-Fu: 5-fluorouracil; DDS: drug delivery system; DEX: dexamethasone; DOX: Doxorubicin; NPs: nanoparticles; Ref: references.

**Table 5:** Summary of studies on XG-based DDSs.

Xanthan Gum (XG)-Based DDSs						
Formulation	Drug Delivery Vehicle	Platform	Disease	Loaded Drug	Observed Effects	Ref.
Carboxyethyl XG-carboxymethyl XG	Hydrogel particle	In vitro and in vivo	Type 2 diabetes	Repaglinide	The system released 97% drug in 4 h. Prolonged drug release for 8 h. Reduction in blood glucose levels in diabetic rats.	[51]
XG	MDFs	In vitro	Type 2 diabetes	Glibenclamide	Instant release of drug. Drug rapid dissolution.Optimum mechanical strength.	[52]
XG	MDFs	In vitro	Hypertension	Amlodipine	Complete drug release within 10 min.	[53]
XG	Gel-AuNPs	In vitro	*P. vulgaris* infection	Hesperidin	Gel showed antimicrobial activity against *P. vulgaris*.	[54]

AuNPs: gold nanoparticles; MDFs: mouth-dissolving films; Ref: references.

**Table 6:** Summary of studies on dextran-based DDSs.

Dextran-Based DDSs						
Formulation	Drug Delivery Vehicle	Platform	Disease	Loaded Drug	Observed Effect	Ref.
Dextran-stabilized perfluorohexane	Ultrasound-responsive nanodroplets	In vitro	Cancer	DOX	Particle size and encapsulation efficiency. Biphasic drug release system of DOX.	[62]
Vinyl-functionalized dextran, vinyl-modified graphene oxide-Laponite	NIR light-responsive hydrogel	Ex vivo	Microbial infection	Ciproflo-xacin	Drug dispersion in NIR-controllable. Antibacterial effect. Good compatibility with blood.	[64]
Modified dextran (dextran-sulfate-PVGLIG)	Nanomicelles	In vitro and in vivo	Rheumatoid arthritis	Cel	High entrapment of drug in nanomicelles. Effectively delivered the drug to the inflammatory joint. Greater anti-RA effects. Lower systemic toxicity in comparison to free Cel.	[65]
Dextran-graft-poly(2-(diisopropylamino) ethyl methacrylate-co-2-(2′,3′,5′-triiodobenzoyl) ethyl methacrylate)	pH-sensitive micelle	In vitro and in vivo	Breast cancer	DOX	Optimally release DOX into MCF-7 cells. Excellent anticancer efficacy. Effectively reduce the growth of the tumor.	[66]

AuNPs: gold nanoparticles; Cel: celastrol; DOX: doxorubicin; Ref: references.
