# Correction: Nordin et al. The State of the Art of Natural Polymer Functionalized Fe_3_O_4_ Magnetic Nanoparticle Composites for Drug Delivery Applications: A Review. *Gels* 2023, *9*, 121

**DOI:** 10.3390/gels10010064

**Published:** 2024-01-15

**Authors:** Abu Hassan Nordin, Zuliahani Ahmad, Siti Muhamad Nur Husna, Rushdan Ahmad Ilyas, Ahmad Khusairi Azemi, Noraznawati Ismail, Muhammad Luqman Nordin, Norzita Ngadi, Nordin Hawa Siti, Walid Nabgan, Abd Samad Norfarhana, Mohammad Saifulddin Mohd Azami

**Affiliations:** 1 Faculty of Chemical and Energy Engineering, Universiti Teknologi Malaysia, Skudai 81310, Johor, Malaysia; abuhassannordin@gmail.com (A.H.N.); norzita@cheme.utm.my (N.N.); farahfarhana.as@gmail.com (A.S.N.); 2 Faculty of Applied Sciences, Universiti Teknologi MARA (UiTM), Arau 02600, Perlis, Malaysia; zuliahani@uitm.edu.my (Z.A.); husna.md94@gmail.com (S.M.N.H.); saifulddin@uitm.edu.my (M.S.M.A.); 3 Centre for Advanced Composite Materials (CACM), Universiti Teknologi Malaysia (UTM), Skudai 81310, Johor, Malaysia; 4 Institute of Marine Biotechnology, Universiti Malaysia Terengganu, Kuala Terengganu 21030, Terengganu, Malaysia; madkucai89@gmail.com; 5 Department of Clinical Studies, Faculty of Veterinary Medicine, Universiti Malaysia Kelantan, Pengkalan Chepa, Kota Bharu 16100, Kelantan, Malaysia; luqman.n@umk.edu.my; 6 Centre for Nanotechnology in Veterinary Medicine (NanoVet), Faculty of Veterinary Medicine, Universiti Malaysia Kelantan, Pengkalan Chepa, Kota Bharu 16100, Kelantan, Malaysia; 7 Pharmacology Unit, School of Basic Medical Sciences, Faculty of Medicine, Universiti Sultan Zainal Abidin, Kuala Terengganu 20400, Terengganu, Malaysia; hawanordin@gmail.com; 8 Departament d’Enginyeria Química, Universitat Rovira I Virgili, Av. Països Catalans 26, 43007 Tarragona, Spain; wnabgan@gmail.com; 9 Department of Petrochemical Engineering, Politeknik Tun Syed Nasir Syed Ismail, Pagoh Education Hub, Pagoh Muar 84600, Johor, Malaysia

In the original publication [1], the Reference 17 (Section 2, Paragraph 1) was wrongly cited [2]. The right reference that the authors intended to cite is [3] by Patel et al. 

With this correction, the reference has been adjusted accordingly. The authors state that the scientific conclusions are unaffected. This correction was approved by the Academic Editor. The original publication has also been updated.

## References

[1] Nordin A.H., Ahmad Z., Husna S.M.N., Ilyas R.A., Azemi A.K., Ismail N., Nordin M.L., Ngadi N., Siti N.H., Nabgan W. (2023). The State of the Art of Natural Polymer Functionalized Fe_3_O_4_ Magnetic Nanoparticle Composites for Drug Delivery Applications: A Review. Gels.

[2] Vickers N.J. (2017). Animal communication: When I’m calling you, will you answer too?. Curr. Biol..

[3] Patel J.K., Patel A.P. (2019). Passive targeting of nanoparticles to cancer. Surface Modification of Nanoparticles for Targeted Drug Delivery.

